# APOE4 Exacerbates Alzheimer‐Like Pathologies and Cognitive Deficits Induced by Blood‐Derived Aβ in a Mouse Model

**DOI:** 10.1111/acel.70205

**Published:** 2025-09-04

**Authors:** Zhong‐Yuan Yu, Xiao‐Yu Liu, Qiong‐Yan Li, Jin‐Mei Tuo, Qi Tan, Zhi‐Hao Liu, Zi‐Yu Yuan, Ru Zeng, Yang Zhao, Jiang‐Hui Li, Yu‐Di Bai, Gui‐Hua Zeng, Dong‐Wan Chen, Xian‐Le Bu, Wang‐Sheng Jin, Yan‐Jiang Wang

**Affiliations:** ^1^ Department of Neurology and Centre for Clinical Neuroscience Daping Hospital, Army Medical University (Third Military Medical University) Chongqing China; ^2^ Institute of Brain and Intelligence Army Medical University (Third Military Medical University) Chongqing China; ^3^ Chongqing Key Laboratory of Ageing and Brain Diseases Chongqing China

**Keywords:** Alzheimer's disease, APOE4, Aβ, bone marrow transplantation, cognitive deficits

## Abstract

Apolipoprotein E4 (APOE4) is a significant risk for both familial Alzheimer's disease (AD) and sporadic AD with elusive mechanisms. Previous studies mainly focused on the role of APOE4 in familial AD, with less attention to sporadic AD. Our previous study demonstrated that blood cell‐derived amyloid‐β (Aβ) can enter the brain and induce AD‐like pathologies, providing a novel animal model to study sporadic AD to a certain extent. The impacts of APOE4 on Alzheimer‐like pathologies and cognitive deficits induced by blood‐derived Aβ remain unknown. In the present study, we found that APOE4 prompted the entry of blood Aβ into the brain. APOE4 recipient mice showed impaired integrity of the blood–brain barrier and higher Aβ levels in the brain after transplantation of bone marrow cells from APP/PS1•APOE4 mice. In addition, we observed that the APOE4 recipient mice displayed aggravated tau hyperphosphorylation, neuronal degeneration, neuroinflammation, and behavioral deficits at the age of 12 months. Our study demonstrates that APOE4 is capable of facilitating the entry of blood‐derived Aβ into the brain and enhancing the AD‐like pathologies triggered by blood‐derived Aβ. Our findings provide a possible way by which APOE4 elevates the risk of sporadic AD.

## Graphical abstract

APOE4 promotes the entry of blood‐derived Aβ into the brain and impairs microglial phagocytosis of Aβ. Additionally, APOE4 elevates cerebral Aβ levels and exacerbates blood‐derived Aβ‐induced cognitive deficits.

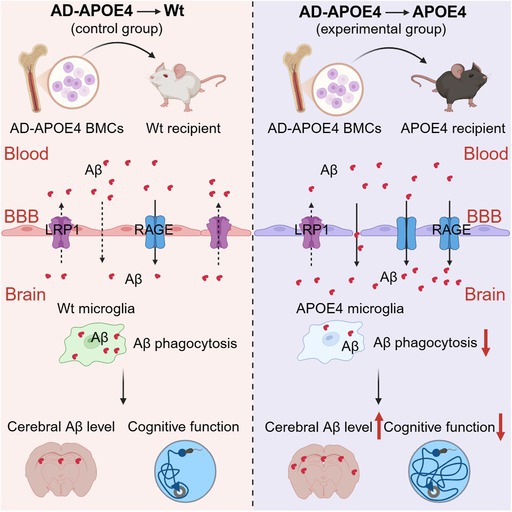

## 1 Introduction

Alzheimer's disease (AD), characterized by a progressive decline in cognitive function and memory impairment, stands as the foremost cause of dementia among the elderly population (Jia et al. 2020). Currently, it impacts approximately 50 million individuals globally, thereby imposing a substantial burden on both society and families (Jia et al. 2018). AD can be categorized into familial AD, constituting 1% of all AD cases, and sporadic AD, which accounts for 99% (Selkoe and Hardy 2016). Familial AD arises from the overexpression of amyloid‐β peptide (Aβ) as a result of mutations in the amyloid precursor protein (APP) and presenilin (PS) 1/2 genes (Selkoe and Hardy 2016). In contrast, the underlying mechanism of sporadic AD, being the most prevalent form of AD, remains elusive. Elucidating the etiology of sporadic AD would be conducive to formulating efficacious preventive strategies against AD.

Apolipoprotein E4 (APOE4) exhibits a strong risk and a close association with the progression of both sporadic AD and familial AD (Martens et al. 2022). In the context of familial AD, APOE4 is capable of modulating the processing of APP, consequently resulting in an elevation of Aβ generation (Huang et al. 2017). Simultaneously, APOE4 might also have an impact on the clearance of Aβ (Sharman et al. 2010). For instance, APOE4 could potentially diminish the phagocytic efficacy of microglia with respect to Aβ (Lin et al. 2018). APOE4 has also been demonstrated to enhance the risk of sporadic AD by 4–16 times, yet the underlying mechanisms remain elusive (Frisoni et al. 2022).

It is traditionally believed that AD is a disease confined to the brain itself, with the pathological Aβ originating from the brain itself. However, besides the brain, peripheral tissues like platelets and fibroblasts are also capable of producing Aβ (Chen et al. 1995; Citron et al. 1994). Our previous studies, in conjunction with that of others, have cumulatively demonstrated that Aβ derived from the blood can infiltrate into the brain and precipitate AD‐type pathologies as well as cognitive impairments (Banerjee et al. 2024; Bu et al. 2018; Singh et al. 2024; Sun et al. 2021). This discovery may, to a certain degree, elucidate the mechanism underlying sporadic AD. Given that APOE4 represents a significant risk factor for sporadic AD, it remains uncertain whether it would affect the roles of blood‐derived Aβ in AD pathogenesis. In the current study, we initially examined the influence of APOE4 on the entry of blood Aβ into the brain by injecting Cy5.5‐labeled Aβ. Subsequently, we transplanted bone marrow cells (BMCs) from AD•APOE4 mice into APOE4 mice to explore the impact of APOE4 on AD‐type pathologies induced by blood‐born Aβ. Our results indicated that APOE4 can aggravate the Alzheimer‐like pathologies and cognitive deficits induced by blood‐derived Aβ.

## 2 Methods and Materials

APPswe/PS1dE9 transgenic mice (APP/PS1, also referred to as AD mice) and humanized APOE4 mice were obtained from the Jackson Laboratory. APP/PS1 harbors a chimeric mouse/human amyloid precursor protein (Mo/HuAPP695swe) and a mutant human presenilin 1 (PS1‐dE9) under the prion promoter. AD mice were crossed with homogeneous APOE4 mice to generate heterozygous AD•APOE4/Wt mice, and then heterozygous AD•APOE4/Wt mice were backcrossed with homogeneous APOE4 mice to obtain AD•APOE4/4 (AD•APOE4) mice. A random number table was employed to conduct random allocation of animals to different treatment groups. To investigate the effect of APOE4 on the entry of blood Aβ into the brain, Cy5.5‐labeled monomeric Aβ42 was intravenously injected into 3‐month‐old male APOE4 and wild type (Wt) mice (*N* = 3 per group); the fluorescence intensity of the brain was measured. Male APOE4 and Wt mice at the age of 3 months were subjected to a microdialysis experiment to collect brain interstitial fluid (ISF) after intravenous human Aβ injection, and the human Aβ level in ISF was determined by enzyme‐linked immunosorbent assay (ELISA) kit (*N* = 4 per group). To explore the effect of APOE4 on the Alzheimer‐like pathologies and cognitive deficits induced by blood‐derived Aβ, 3‐month‐old Wt and APOE4 mice (*N* = 12 per group) were irradiated at a dose of 8.5 Gy with a lead block covering the brain to prevent brain damage (Sun et al. 2021). To keep the genetic profiles of blood cells identical and the capacity of blood cells to produce Aβ, both irradiated Wt and APOE4 mice were transplanted with the BMCs from age‐ and sex‐matched APP/PS1 mice carrying homogeneous human APOE4 gene. The male and female mice were balanced for use in this experiment. According to previous studies (Singh et al. 2024; Sun et al. 2021), there might be AD‐like pathologies in the brain at least 9‐month‐post transplantation with BMCs from AD mice; thus, we measured the human Aβ levels in blood and brain at the age of 12 months. In addition, other AD‐related pathologies, including neuroinflammation, neurodegeneration, blood–brain barrier (BBB) integrity, and phosphorylation of tau, as well as cognitive functions were also investigated at the age of 12 months. The detailed methods and materials were presented in the Supporting Information.

## 3 Results

### 3.1 APOE4 Facilitated Blood Aβ Entering the Brain

**FIGURE 1:**
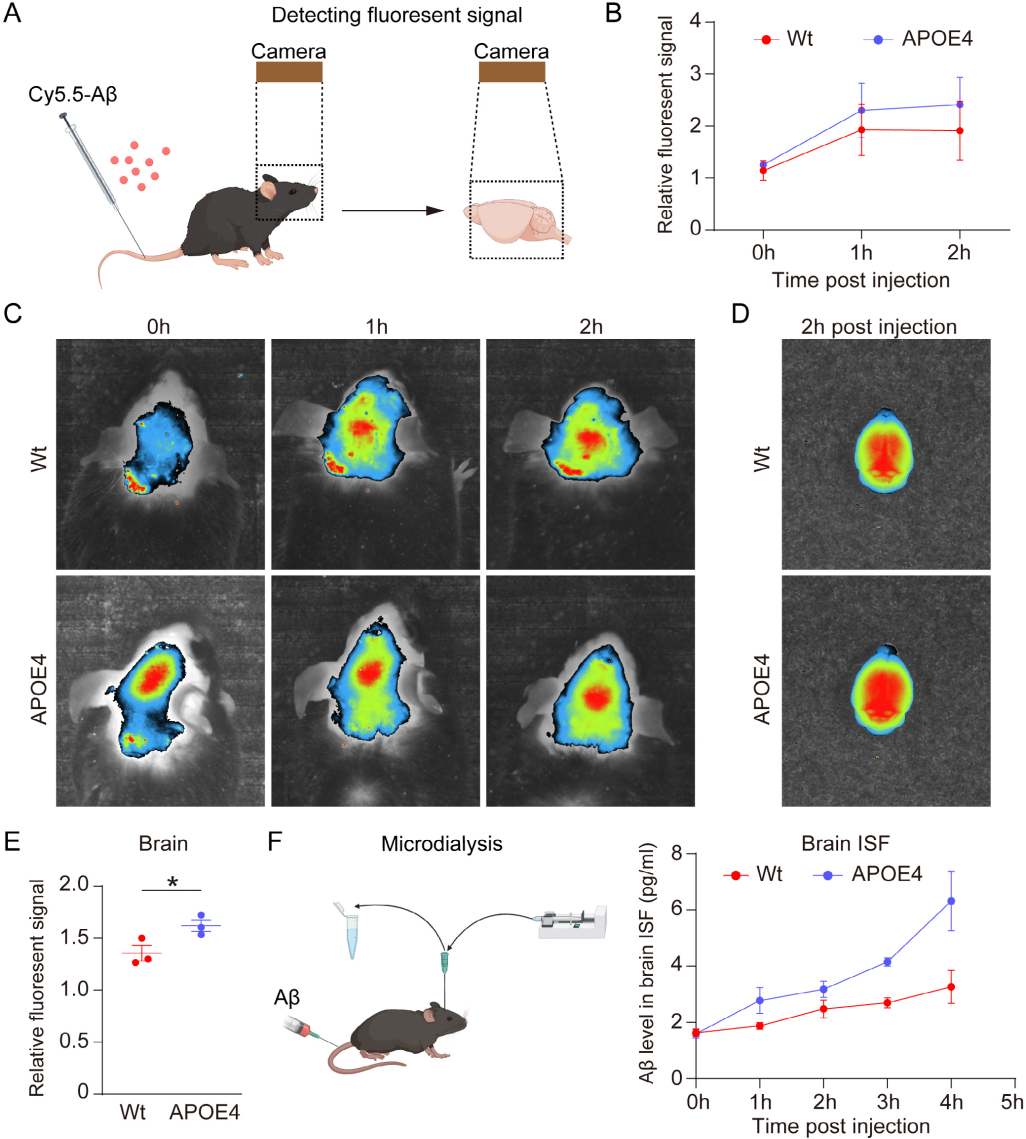
Effect of APOE4 on the entry of blood Aβ into the brain. (A) Graphic diagram of investigating the entry of blood Aβ into the brain. (B, C). The distribution and dynamic changes of Cy5.5‐labeled Aβ in the brain region over time via in vivo near‐infrared imaging (*N* = 3). (D) Representative fluorescent images of brain 2 h after the injection of Cy5.5‐labeled Aβ (*N* = 3). (E) Comparisons of relative fluorescent signal cerebral parenchymal between Wt and APOE4 mice. (F) Graphic diagram of microdialysis and comparisons of human Aβ level in brain interstitial fluid between APOE4 and Wt mice (*N* = 4). *p < 0.05.

To explore the impact of APOE4 on the entry of blood Aβ into the brain, we injected Cy5.5‐labeled human Aβ into APOE4 and Wt mice via the tail vein (Figure 1A). We found that the relative fluorescence intensity in the brain region area was higher in the APOE4 mice than in the Wt mice (Figure 1B,C). To exclude the interference of the vessels on the scalp, we carefully dissected the brain and examined the fluorescence intensity 2 h after injection. We found a higher relative fluorescence intensity of the brain in APOE4 mice compared to that in Wt mice (*p* = 0.0445, Figure 1E). In addition, we found a higher human Aβ level in the ISF in APOE4 mice compared to that in Wt mice after intravenous human Aβ injection (Figure 1F). These results collectively indicate that APOE4 promotes blood Aβ entering the brain.

### 3.2 APOE4 Elevated the Levels of Blood‐Derived Aβ in the Brain

**FIGURE 2:**
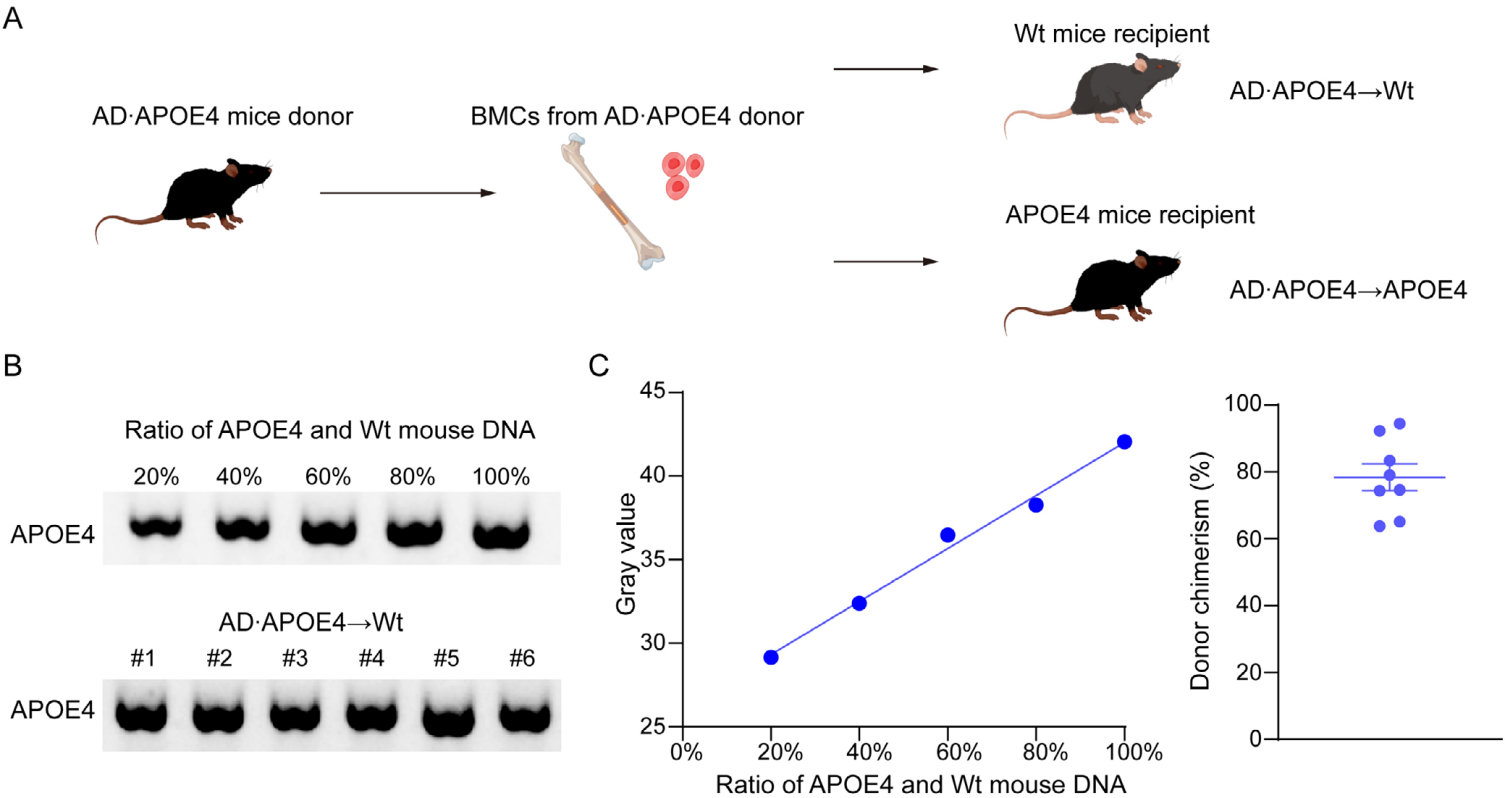
Investigating blood chimerism in APOE4 recipient mice after bone marrow transplantation. (A) Graphic diagram of bone marrow transplantation strategy. (B) The gel image of human APOE4 gene PCR products amplified from the mixture of APOE4 and Wt DNA as well as the DNA sample from the APOE4 recipient mice. (C) The fitting curve of the gray value of human APOE4 gene PCR products and the ratio of human APOE4 and Wt mouse DNA. (D) The level of donor marrow engraftment in the AD•APOE4 → Wt mice (*N* = 8). AD•APOE4 → Wt mice denotes that Wt mice received bone marrow cells from APP/PS1 mice carrying humanized APOE4 gene.

**FIGURE 3:**
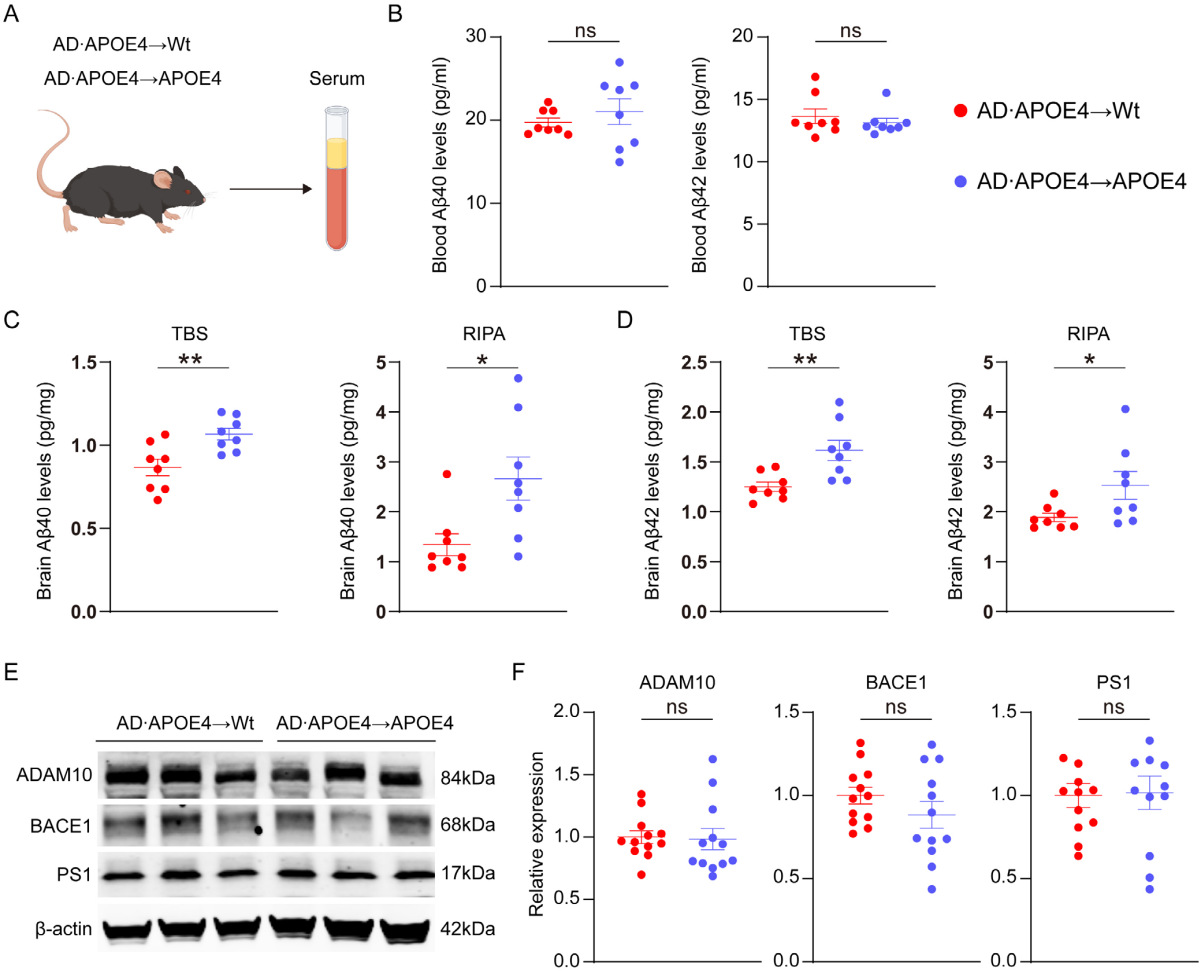
Effect of APOE4 on blood and cerebral Aβ levels after bone marrow transplantation. (A) Graphic diagram of examining the blood Aβ levels in AD•APOE4 → Wt mice and AD•APOE4 → APOE4 mice. (B) Comparisons of blood Aβ40 and Aβ42 levels between AD•APOE4 → Wt mice and AD•APOE4 → APOE4 mice (*N* = 8). (C) Comparisons of brain Aβ40 levels in TBS and RIPA solution between AD•APOE4 → Wt mice and AD•APOE4 → APOE4 mice (*N* = 8). (D) Comparisons of brain Aβ42 levels in TBS and RIPA solution between AD•APOE4 → Wt mice and AD•APOE4 → APOE4 mice (*N* = 8). (E) Representative western‐blotting images of Aβ generation‐related enzymes. (F) Comparisons of levels of Aβ generation‐related enzymes (ADAM10, BACE1, and PS1) between AD•APOE4 → Wt mice and AD•APOE4 → APOE4 mice (*N* = 12). *p < 0.05; **p < 0.01; AD•APOE4 → Wt mice denotes that Wt mice received bone marrow cells from APP/PS1 mice carrying humanized APOE4 gene. AD•APOE4 → APOE4 mice denotes that APOE4 mice received bone marrow cells from APP/PS1 mice carrying humanized APOE4 gene.

To investigate whether APOE4 would influence the levels of blood‐derived Aβ in the brain under the long‐term condition, we transferred the BMCs from AD•APOE4 mice that express the human Aβ into the APOE4 recipient mice (Figure 2A). The donor engraftment of blood cells derived from transplanted BMCs in the APOE4 recipient mice was about 80% (Figure 2B,C). We collected the serum from the recipient mice at the age of 12 months and examined the Aβ40 and Aβ42 levels (Figure 3A). There were no significant differences in blood Aβ40 levels between AD•APOE4 → Wt mice and AD•APOE4 → APOE4 mice (*p* = 0.44 for Aβ40, *p* = 0.46 for Aβ42; Figure 3B). Next, we extracted the brain Aβ using Tris‐buffered saline (TBS) and Radio‐immunoprecipitation assay (RIPA) solution. In comparison to AD•APOE4 → Wt mice, AD•APOE4 → APOE4 mice had higher levels of human Aβ40 in TBS and RIPA solution (*p* = 0.0055, *p* = 0.0162; Figure 3C) and human Aβ42 in TBS solution and RIPA solution (*p* = 0.0053, *p* = 0.0449; Figure 3D). Unfortunately, we found that there were no obvious Aβ plaques in both AD•APOE4 → APOE4 mice and AD•APOE4 → Wt mice (Figure S1).

To investigate whether blood‐derived Aβ would influence the expressions of endogenous Aβ production‐associated enzymes in the brain, we examined the levels of a disintegrin and metallopeptidase domain 10 (ADAM10), β‐site amyloid precursor protein‐cleaving enzyme 1 (BACE1) and PS1 in the recipient mice. Interestingly, we discovered that there were no significant disparities in the levels of ADAM10, BACE1, and PS1 between AD•APOE4 → Wt mice and AD•APOE4 → APOE4 mice (Figure 3E,F). To reveal the impact of APOE4 on microglial Aβ clearance, we isolated primary microglia from Wt and APOE4 mice and cocultured them with FITC‐labeled Aβ. We found a compromised microglial Aβ phagocytosis in microglia from APOE4 mice compared to Wt mice (Figure S2). Collectively, our results indicate that APOE4 elevates the level of blood‐derived Aβ in the brain.

### 3.3 APOE4 Impaired the BBB Integrity and Elevated Aβ Transporter Levels After BMT

**FIGURE 4:**
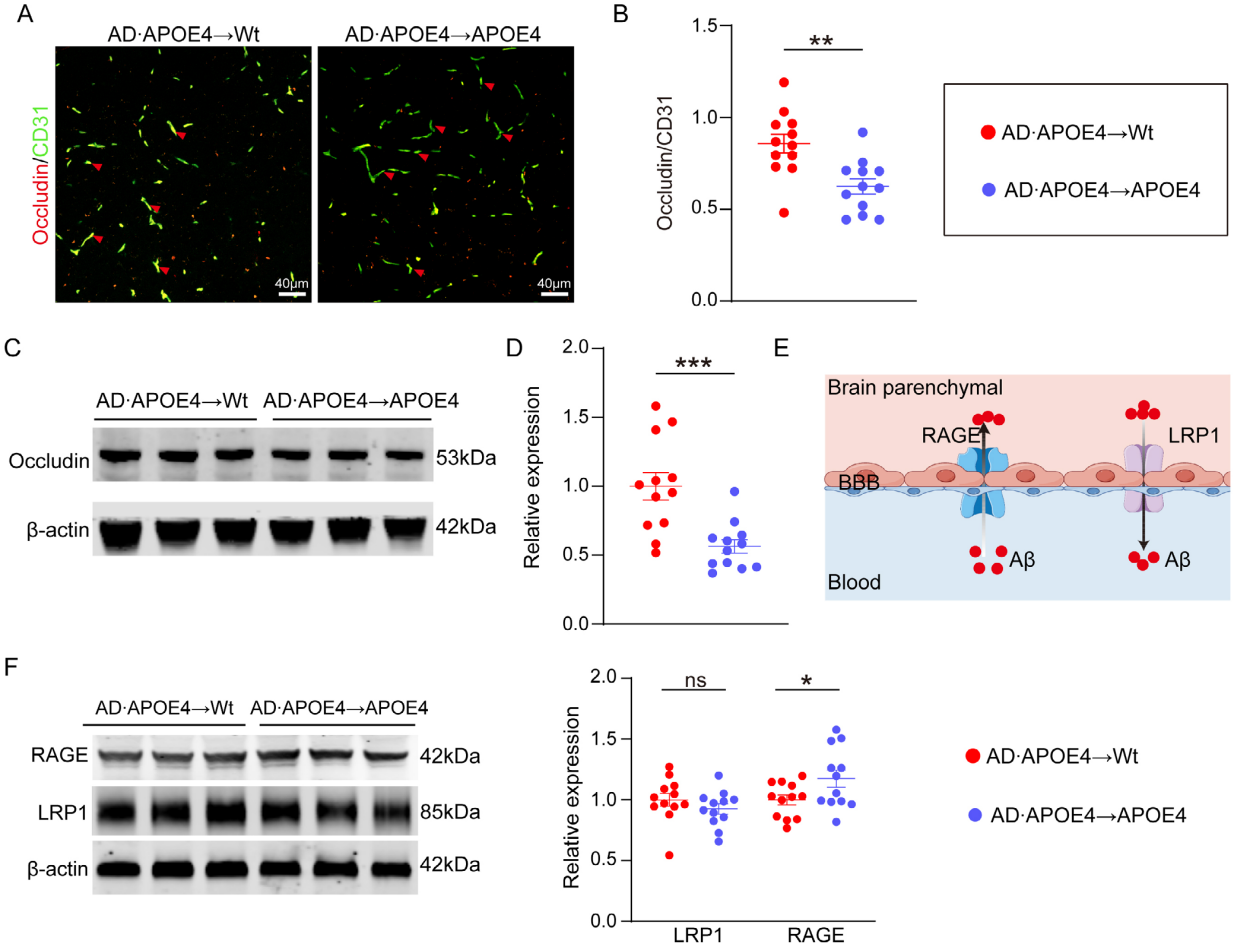
Effect of APOE4 on BBB integrity after bone marrow transplantation. (A) Representative fluorescent images of Occludin and CD31 staining. (B) Comparison of relative Occludin expression on the CD31+ vessels between AD•APOE4 → Wt mice and AD•APOE4 → APOE4 mice (*N* = 12). (C) Representative western‐blotting images of Occludin expression in the brain. (D) Comparison of relative expression of cerebral Occludin between AD•APOE4 → Wt mice and AD•APOE4 → APOE4 mice (*N* = 12). (E) Graphic diagram of the role of RAGE and LRP1 in transporting Aβ. (F) Representative western‐blotting images and comparison of relative Occludin expression between AD•APOE4 → Wt mice and AD•APOE4 → APOE4 mice (*N* = 12). *p < 0.05; **p < 0.01; ***p < 0.001; Scale bar in A is 20 μm. AD•APOE4 → Wt mice denotes that Wt mice received bone marrow cells from APP/PS1 mice carrying humanized APOE4 gene. AD•APOE4 → APOE4 mice denotes that APOE4 mice received bone marrow cells from APP/PS1 mice carrying humanized APOE4 gene. LRP1 denotes LDL receptor related protein 1; RAGE denotes the receptor of advanced glycation end products.

As BBB integrity plays a crucial role in maintaining the balance of Aβ levels between blood and brain, we compared the expressions of Occludin, which is a marker for BBB integrity, between AD•APOE4 → Wt mice and AD•APOE4 → APOE4 mice. We found a lower Occludin expression (normalized to CD31, a marker for vascular endothelial cells) in AD•APOE4 → APOE4 mice compared to AD•APOE4 → Wt mice (*p* = 0.0019, Figure 4A,B). The results of western blotting also revealed a lower level of occludin in AD•APOE4 → APOE4 mice (*p* = 0.0007, Figure 4C,D). In addition, we also investigated expressions of Aβ transporter in the brain. Low‐density lipoprotein receptor‐related protein 1 (LRP1) can transport Aβ from the brain into blood, and Receptor for advanced glycation end products (RAGE) can transport Aβ from the blood into brain (Figure 4E). Interestingly, we found a decreased expression of LRP1 in AD•APOE4 → APOE4 mice (Figure 4F), although the difference did not reach statistical significance. AD•APOE4 → APOE4 mice had a lower level of RAGE compared to AD•APOE4 → Wt mice (*p* = 0.0453, Figure 4F). Overall, our study indicates that APOE4 impairs the integrity of BBB and elevates levels of Aβ transporters after BMT.

### 3.4 APOE4 Augmented the Astrogliosis and Microgliosis Induced by Blood‐Derived Aβ

**FIGURE 5:**
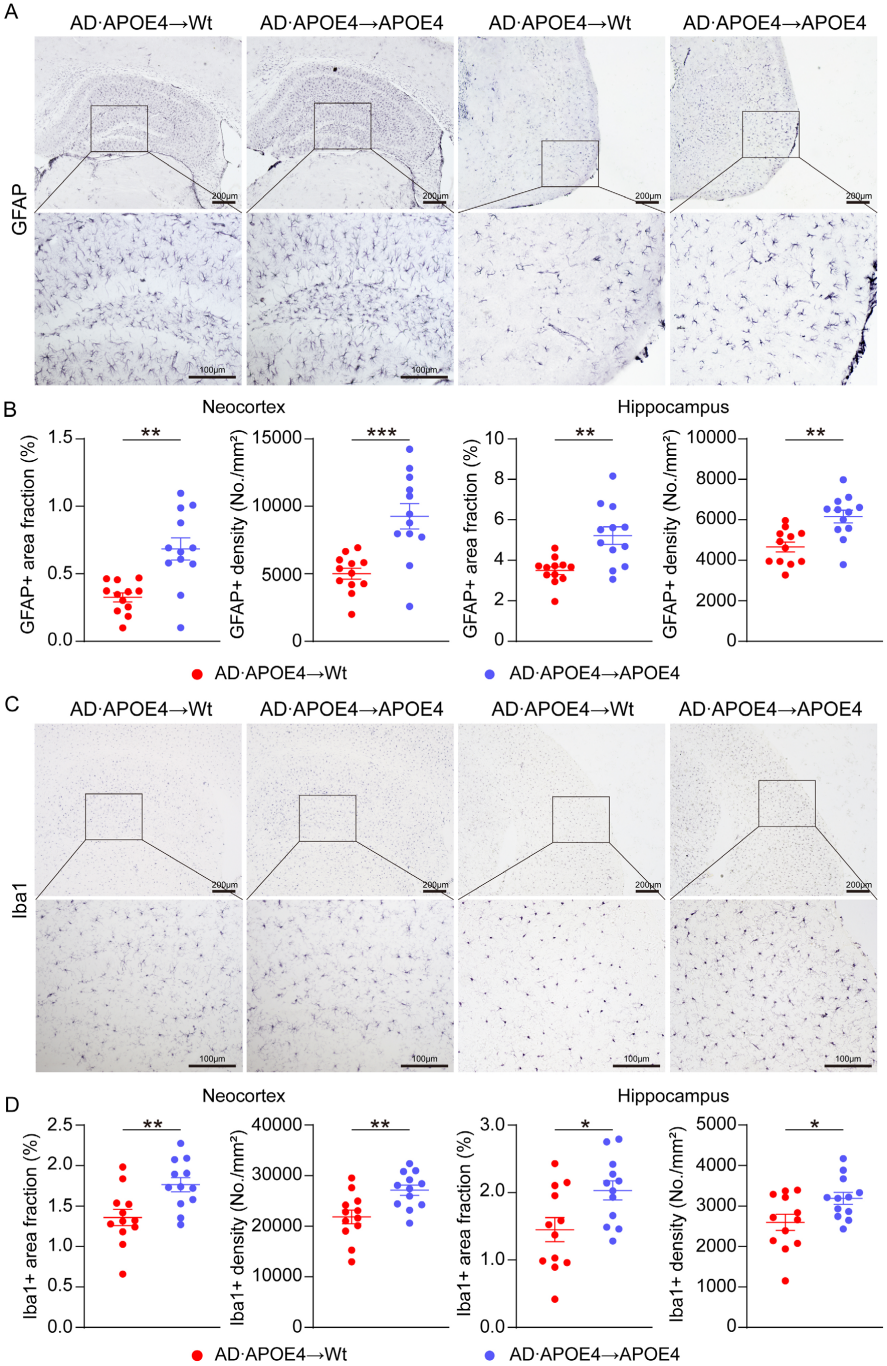
APOE4 augmented astrogliosis induced by blood‐derived Aβ. (A) Representative immunochemistry images of GFAP+ staining. (B) Comparisons of area fraction and density of GFAP+ staining in neocortex and hippocampus between AD•APOE4 → Wt mice and AD•APOE4 → APOE4 mice (*N* = 12). (C) Representative immunochemistry images of Iba1+ staining. (D) Comparisons of area fraction and density of Iba1+ staining in neocortex and hippocampus between AD•APOE4 → Wt mice and AD•APOE4 → APOE4 mice (*N* = 12). *p < 0.05; **p < 0.01; ***p < 0.001. Scale bar in A is 200 μm (top) and 100 μm (bottom). Scale bar in C is 200 μm (top) and 100 μm (bottom). AD•APOE4 → Wt mice denotes that Wt mice received bone marrow cells from APP/PS1 mice carrying humanized APOE4 gene. AD•APOE4 → APOE4 mice denotes that APOE4 mice received bone marrow cells from APP/PS1 mice carrying humanized APOE4 gene.

Neuroinflammation represents the secondary pathological occurrences following Aβ stimulation. In order to assess the impact of APOE4 on neuroinflammation after BMT, we carried out Glial fibrillary acidic protein positive (GFAP+) staining and Ionized calcium–binding adapter molecule 1 positive (Iba1+) staining to ascertain the levels of astrogliosis and microgliosis. As depicted in Figure 5, in comparison to AD•APOE4 → Wt mice, AD•APOE4 → APOE4 mice exhibited a larger area fraction and a higher density of GFAP+ cells in both the neocortex (*p* = 0.0005 and *p* = 0.0004) and hippocampus (Figure 5A,B). Additionally, it was also determined that AD•APOE4 → APOE4 mice possessed a greater number and density of Iba1+ cells in both the neocortex and hippocampus (*p* = 0.0013 and *p* = 0.0011) relative to AD•APOE4 → Wt mice (Figure 5C,D). In addition, we found that the area fractions of microgliosis and astrogliosis in AD•APOE4 → APOE4 and AD•APOE4 → Wt mice were increased compared to that in APOE4 and Wt mice, respectively (Figure S3). Taken together, our study indicates that APOE4 aggravates the neuroinflammation mediated by blood‐derived Aβ.

### 3.5 APOE4 Exacerbated the Neurodegeneration Induced by Blood‐Derived Aβ

**FIGURE 6:**
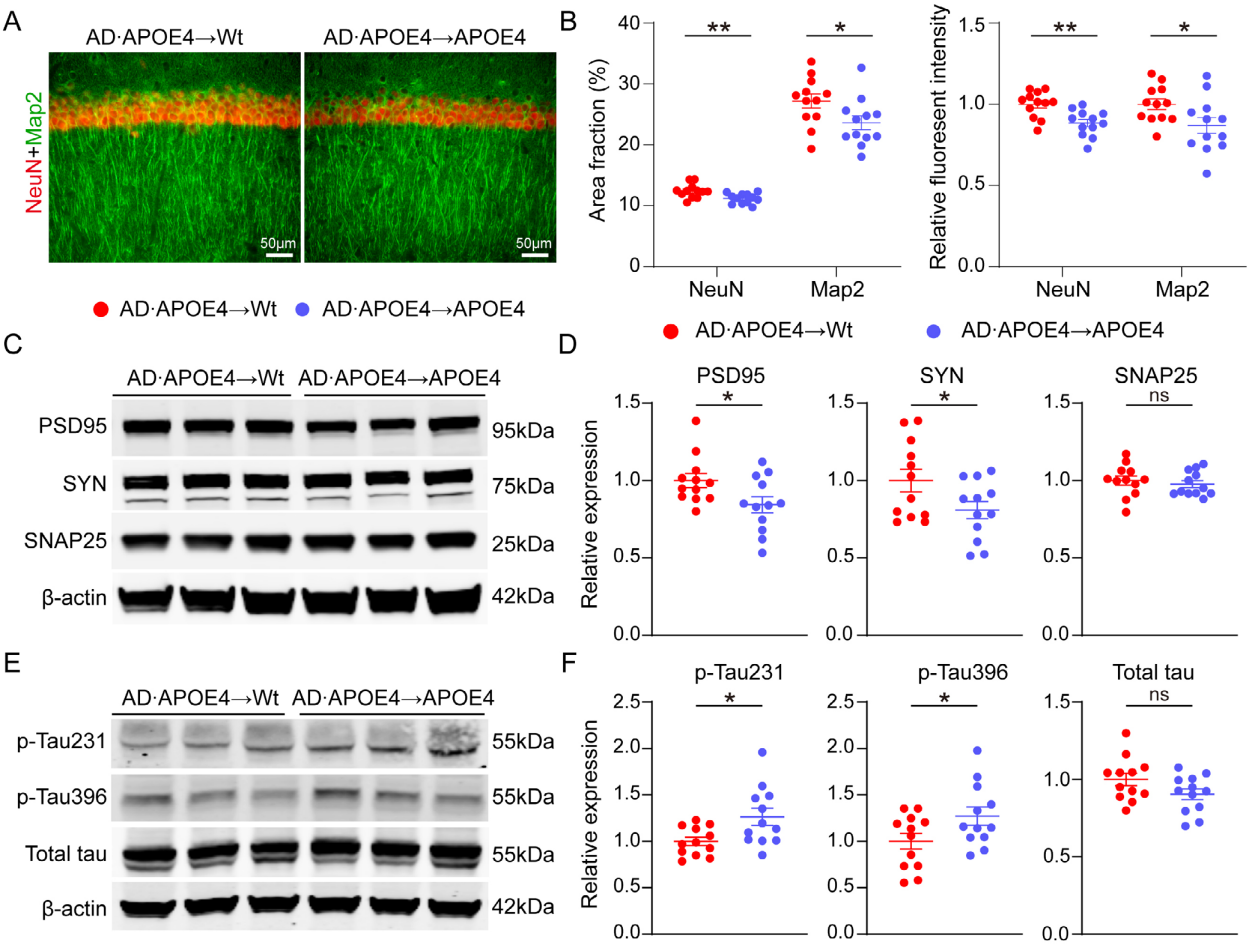
APOE4 exacerbated neurodegeneration induced by blood‐derived Aβ. (A) Representative immunofluorescent images of NeuN and Map2 staining. (B) Comparisons of area fraction and relative fluorescent density of I NeuN+ and Map2+ staining in the CA1 region of hippocampus between AD•APOE4 → Wt mice and AD•APOE4 → APOE4 mice (*N* = 12). (C, D) Representative western‐blotting images and comparisons of the levels of synapse‐related proteins (PSD95, SYN, and SNAP25) between AD•APOE4 → Wt mice and AD•APOE4 → APOE4 mice (*N* = 12). (E, F) Representative western‐blotting images and comparisons of the levels of phosphorylated‐Tau and total Tau proteins between AD•APOE4 → Wt mice and AD•APOE4 → APOE4 mice (*N* = 12). *p < 0.05; **p < 0.01; Scale bar in A is 50 μm. PSD95 denotes postsynaptic density protein 95; SYN denotes synapsin; SNAP‐25 denotes synaptosomal‐associated protein 25. AD•APOE4 → Wt mice denotes that Wt mice received bone marrow cells from APP/PS1 mice carrying humanized APOE4 gene. AD•APOE4 → APOE4 mice denotes that APOE4 mice received bone marrow cells from APP/PS1 mice carrying humanized APOE4 gene.

Neurodegeneration is commonly observed in AD patients, which is manifested as neuron loss and hyperphosphorylated Tau. We examined the number of neurons and the density of dendrites in the hippocampus subsequent to BMT. It was found that in AD•APOE4 → APOE4 mice, the area fractions of Neuronal nuclei positive (NeuN+) cells (*p* = 0.0065) and Microtubule‐associated protein 2 positive (Map2+) dendrites (*p* = 0.0378) were smaller, and the fluorescent intensities of NeuN (*p* = 0.0019) and Map2 (*p* = 0.0373) were lower when compared to AD•APOE4 → Wt mice (Figure 6A,B). Further analysis of synaptic proteins demonstrated that AD•APOE4 → APOE4 mice exhibited lower protein expressions of Postsynaptic density protein 95 (PSD95) (*p* = 0.0344) and Synapsin (SYN) (*p* = 0.0487) in contrast to AD•APOE4 → Wt mice (Figure 6C,D). We also explored the degree of Tau hyperphosphorylation, which is another hallmark of AD, after BMT. Compared to AD•APOE4 → Wt mice, AD•APOE4 → APOE4 mice presented higher levels of Phosphorylated Tau at the Threonine 231 residue (p‐Tau231) (*p* = 0.0164) and Phosphorylated Tau at the Serine 396 residue (p‐Tau396) (*p* = 0.0471), while the total tau levels remained unchanged (Figure 6E,F). Collectively, our results suggest that APOE4 exacerbates the loss of neurons and synaptic proteins as well as the phosphorylation of Tau induced by blood‐derived Aβ.

### 3.6 APOE4 Aggregated Cognitive Deficits Induced by Blood‐Derived Aβ

**FIGURE 7:**
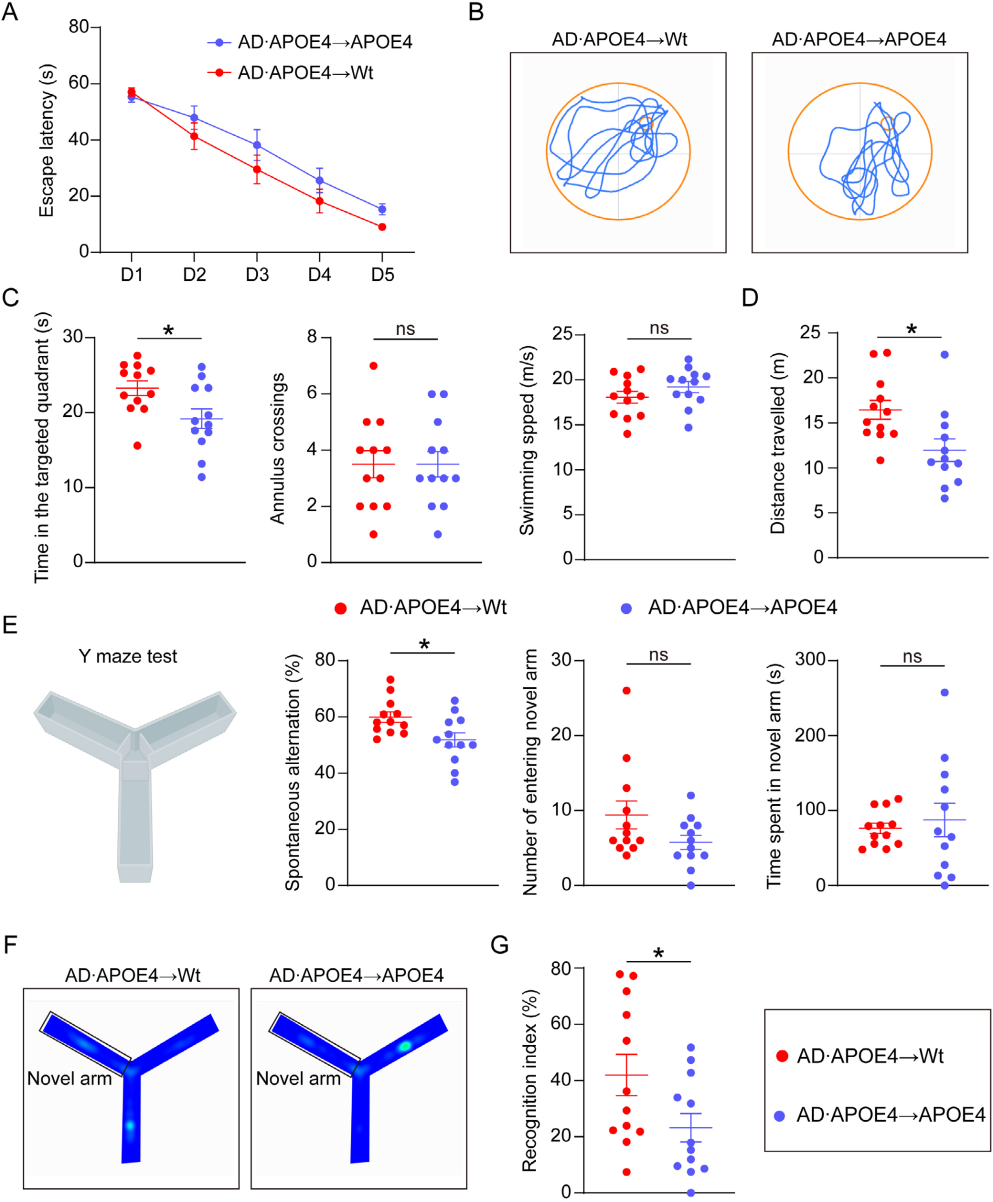
APOE4 aggregated cognitive deficits induced by blood‐derived Aβ. (A) Comparison of escape latency between AD•APOE4 → Wt mice and AD•APOE4 → APOE4 mice in MWM test. (B) Representative images of tracking paths in probe test in MWM test. (C) Comparisons of time spent in the targeted quadrant, annulus crossings, and swimming speed between AD•APOE4 → Wt mice and AD•APOE4 → APOE4 mice in MWM test (*N* = 12). (D) Comparison of distance traveled between AD•APOE4 → Wt mice and AD•APOE4 → APOE4 mice in the open‐field test (*N* = 12). (E) Graphic diagram of Y maze test and the comparisons of spontaneous alternation, number of entering the novel arm and time spent in the novel arm between AD•APOE4 → Wt mice and AD•APOE4 → APOE4 mice in the Y maze test (*N* = 12). (F) Heat map of tracking in the part of novel arm recognition in the Y maze test. (G) Statistics of recognition index between AD•APOE4 → Wt mice and AD•APOE4 → APOE4 mice in the novel object recognition test (*N* = 12). *p < 0.05. MWM test denotes Morris water maze test. AD•APOE4 → Wt mice denotes that Wt mice received bone marrow cells from APP/PS1 mice carrying humanized APOE4 gene. AD•APOE4 → APOE4 mice denotes that APOE4 mice received bone marrow cells from APP/PS1 mice carrying humanized APOE4 gene.

To evaluate the impact of APOE4 on the cognitive performances following BMT, we performed Morris Water Maze (MWM) test, Y maze test, open–field test, and Novel Object Recognition (NOR) test. In the MWM test, AD•APOE4 → APOE4 mice exhibited a delayed escape latency in locating the platform during the training session (Figure 7A). AD•APOE4 → APOE4 mice spent less time exploring the target quadrant (*p* = 0.0210) and had a comparable number of annulus crossings at the platform location (*p* > 0.999), with an unchanged swimming speed when compared to AD•APOE4 → Wt mice (*p* = 0.224) (Figure 7B,C). These results suggest that APOE4 exacerbates the impairment of spatial memory and learning ability induced by blood‐derived Aβ. Moreover, the traveling distance was shorter in the AD•APOE4 → APOE4 group compared to the AD•APOE4 → Wt group (*p* = 0.0118, Figure 7D). In the Y maze test, we observed a lower spontaneous alternation rate in the AD•APOE4 → APOE4 group (*p* = 0.0167, Figure 7E). Additionally, there were no significant differences in the number of entries into the novel arm and the time spent in the novel arm between AD•APOE4 → Wt mice and AD•APOE4 → APOE4 mice (*p* = 0.145, *p* = 0.635; Figure 7E,F). In the NOR test, a lower recognition index was detected in the AD•APOE4 → APOE4 mice compared to the AD•APOE4 → Wt mice (*p* = 0.0468, Figure 7G).

## 4 Discussion

In the current study, our objective was to explore the influence of APOE4 on the AD‐like pathology induced by blood‐derived Aβ. It was determined that APOE4 enhanced the entry of blood Aβ into the brain. In the BMT mouse model, a higher level of the receptor for RAGE, which is accountable for transporting Aβ from blood into the brain parenchyma, was detected in AD•APOE4 → APOE4 mice in comparison to AD•APOE4 → Wt mice. Moreover, AD•APOE4 → APOE4 mice exhibited elevated Aβ levels, intensified microgliosis and astrogliosis, as well as aggravated neurodegeneration in comparison to AD•APOE4 → Wt mice.

The results of our study hold significant value in understanding the role of APOE4 in the pathogenesis of sporadic AD. It has been demonstrated that APOE4 can increase the risk of sporadic AD by 4–16 times (Kim et al. 2009); however, the underlying mechanism remains elusive. To unravel the relevant mechanism, previous studies crossed the APOE4 mouse with a familial AD animal model characterized by Aβ overexpression in the brain (Xiong et al. 2021). Nevertheless, such an animal model fails to elucidate the role of APOE4 in the development of sporadic AD, as sporadic AD is not caused by Aβ overexpression in the brain. Consequently, the search for a sporadic AD model would be conducive to identifying the role of APOE4 in the progression of this disease. Traditionally, it is believed that cerebral Aβ is generated through the proteolytic processing of APP in neurons (Karran et al. 2011). However, Aβ can also be produced in platelets (Chen et al. 1995), skeletal muscle (Kuo et al. 2000), and vascular walls (Davies et al. 2000) provided that the necessary components (APP and relevant enzymes) for Aβ production are present in those tissues. Moreover, previous studies have suggested that blood‐born Aβ can enter the brain and trigger AD‐like pathologies (Bu et al. 2018; Singh et al. 2024; Sun et al. 2021). To a certain extent, the blood Aβ‐induced AD mouse model might be regarded as a sporadic AD mouse model for the following two reasons: (1) There is no Aβ overexpression in the brain in this mouse model; (2) although Aβ is overproduced in blood cells, it takes a long period (at least 12 months of age) for this mouse model to start developing AD pathologies. In this context, our study discovered that APOE4 can facilitate the entry of Aβ into the brain, as evidenced by the results of ELISA and immunochemistry. Additionally, we also found that APOE4 intensifies the AD‐like pathologies and cognitive dysfunction induced by blood‐derived Aβ. It is needed to point out that there was no obvious Aβ deposition in the brain in both Wt recipient and APOE4 recipient mice, which is different from our previous study that blood‐derived Aβ can deposit in the brain parenchymal in BMT model (Sun et al. 2021). Compared to the prior study (Sun et al. 2021), we protected the brain using a lead block from radiation‐induced damage, including BBB damage, glial dysfunctions, etc. The damage would help blood‐derived Aβ to deposit in the brain, and this might be a possible reason for the difference in the Aβ deposition. Overall, our findings not only broaden the understanding that blood‐derived Aβ can induce AD‐type pathologies but also uncover a possible role of APOE4 in the pathogenesis of sporadic AD.

The mechanisms by which APOE4 promotes the entry of blood Aβ into the brain and exacerbates blood Aβ‐induced AD‐like pathologies require further clarification. There exists a dynamic equilibrium of Aβ between the brain and the periphery, which is maintained with the assistance of BBB, blood‐cerebrospinal fluid barrier, arachnoid villi, and glymphatic‐lymphatic pathway (Wang et al. 2017). The BBB integrity is crucial for safeguarding brain cells against exogenous substances and preserving the physiological functions of the brain. Our study revealed that the integrity of the BBB was compromised in APOE4 recipient mice, thereby providing the potential for exogenous substances to infiltrate the brain. To address this issue, we intravenously injected Cy5.5 labeled‐Aβ into the APOE4 mice and detected a greater intensity of positive signals in the brains of APOE4 mice. This finding suggests that the APOE4‐mediated impairment of the BBB may facilitate the entry of blood Aβ into the brain. The LRP1 and RAGE on the BBB play significant roles in transporting Aβ out of and into the brain, respectively (Deane et al. 2009; Li et al. 2025). In our BMT mouse model, we observed that APOE4 recipient mice exhibited remarkably higher levels of RAGE and slightly lower levels of LRP1 compared to Wt recipient mice. This may potentially account for the mechanism by which APOE4 promotes the entry of blood‐derived Aβ into the brain. This notion was previously demonstrated that APOE4 impairs LRP1‐mediated Aβ clearance on the BBB (Deane et al. 2008; Ma et al. 2018). Furthermore, the impaired clearance of Aβ within the brain significantly contributes to the progression of AD (Roberts et al. 2017). Microglia serve as the primary immune cells responsible for clearing cerebral Aβ. Prior studies have demonstrated that very few blood‐immune cells infiltrated into the brain after BMT unless microglia are ablated prior to BMT; thus, we reason that the local genotype of microglia did not change (Sun et al. 2024; Xu et al. 2020; Yu et al. 2025). Our findings in combination with others demonstrated that APOE4 impairs the microglial clearance of Aβ (Zhao et al. 2014). The reduced microglial Aβ uptake induced by APOE4 may also potentially contribute to the manifestation of AD‐like pathologies in the presence of blood Aβ stimulation.

There were some limitations in our study. First, we did not provide the immunochemistry evidence showing the blood‐derived Aβ in the brain parenchyma, as it is hard to label undeposited (soluble) Aβ species using the immunochemistry approach. Alternatively, we performed the microdialysis and ELISA experiment to measure the blood‐derived Aβ level in brain ISF and found a clear increase in Aβ level in ISF after intravenous Aβ injection, demonstrating the entry of blood Aβ into the brain parenchyma. Second, the mouse brain was covered with a lead block to prevent brain damage during irradiation. Although this procedure avoids brain damage, shielding the brain also shields myeloid progenitor cells in the skull bone marrow and brain vasculature, resulting in incomplete ablation of hematopoietic stem cells in the periphery. Third, the APOE4 mice are constructed by knocking in the human APOE4 gene; thus, the ideal control mice should be the humanized APOE3 mice rather than Wt mice. Further study should be conducted to validate the findings in APOE3 mice. Fourth, while we observed that APOE4 exacerbates blood‐derived Aβ‐induced gliosis, our claim that APOE4 augments the associated neuroinflammation is limited by the lack of inflammatory cytokine measurements, and their determinations would strengthen this notion.

In conclusion, our study clearly demonstrates that APOE4 is capable of facilitating the entry of blood‐borne Aβ into the brain and enhancing the AD‐like pathologies triggered by blood‐derived Aβ. This finding offers a possible way by which APOE4 elevates the risk of sporadic AD.

## Author Contributions

Yan‐Jiang Wang conceived and designed the project. Zhong‐Yuan Yu, Xiao‐Yu Liu, Qiong‐Yan Li, Jin‐Mei Tuo, Qi Tan, Zhi‐Hao Liu, Zi‐Yu Yuan, Ru Zeng, Yang Zhao, and Jiang‐Hui Li conducted experiments. Zhong‐Yuan Yu, Xiao‐Yu Liu, and Qiong‐Yan Li analyzed and interpreted data. Zhong‐Yuan Yu and Yan‐Jiang Wang wrote the manuscript. All authors discussed the results and commented on the manuscript.

## Conflicts of Interest

The authors declare no conflicts of interest.

## Supporting information


**Figure S1:** acel70205‐sup‐0001‐Figures.docx.

## Data Availability

The data that support the findings of this study are available from the corresponding author upon reasonable request.
