# Supplementary material for: APOE4 Exacerbates Alzheimer‐Like Pathologies and Cognitive Deficits Induced by Blood‐Derived Aβ in a Mouse Model
Source: Aging Cell. 2025 Sep 4;24(10):e70205. doi: 10.1111/acel.70205 (PMC12507402; doi:10.1111/acel.70205)
Supplement: Supplementary file 1 — Figure S1: acel70205‐sup‐0001‐Figures.docx. [file ACEL-24-e70205-s001.docx]

**Supplementary methods and materials**

**Animals**

APPswe/PS1dE9 transgenic mice (APP/PS1, also referred to as AD mice) and humanized APOE4 mice were obtained from the Jackson Laboratory (Bar Harbor, MA, USA; JAX catalogue number: 005864 for APP/PS1 mice and 027894 for APOE4 mice). APP/PS1 harbors a chimeric mouse/human amyloid precursor protein (Mo/HuAPP695swe) and a mutant human presenilin 1 (PS1-dE9) under the prion promotor. C57BL/6J wild type (Wt) mice were provided by the animal laboratory of the Third Military Medical University. These genetically modified mice shared the same genetic background. AD mice were crossed with homogeneous APOE4 mice to generate heterozygous AD⬝APOE4/Wt mice, and then heterozygous AD-APOE4/Wt mice were backcrossed with homogeneous APOE4 mice to obtain AD⬝APOE4/4 (AD⬝APOE4) mice. Female and male mice were balanced used. A random number table was employed to conduct random allocation of animals to different treatment groups. Our experimental procedures complied with relevant ethical standards and regulations. Our study was approved by the Laboratory Animal Welfare and Ethics Committee of Daping Hospital affiliated to the Army Medical University (Third Military Medical University).

**In vivo optical near‑infrared imaging of blood Aβ entering the brain**

Three-month-old male mice underwent surgical scalp preparation and fasted 24 h in advance to avoid fluorescent signal interference by hair. Cy5.5-labeled monomeric Aβ42 (200μL, 0.1g/mL) was intravenously injected to mice via tail vein. Then, the fluorescence intensity of the brain area of mice was dynamically detected every 1 h. In addition, the fluorescence intensity of the brain was also measured 2 hours after injection. Near-infrared imaging was performed on a FUSION FX7 EDGE Imaging System. The data were analyzed by EvolutionCapt software (V17.03). The relative fluorescent signal was normalized to the initial condition before the injection of Cy5.5-labeled Aβ.

**Bone marrow transplantation (BMT)**

BMT was performed according to in accordance with our prior study (Yu, Xu, et al., 2022). Briefly, 3-month-old Wt and APOE4 mice were subjected to irradiation at a dose of 8.5 Gy (at a rate of approximately 0.4 Gy per minute) using a radioactive source with cobalt (Co) as the principal radioactive constituent. The dose of 8.5 Gy was chosen as it not only obtains high chimeric rate of blood cells but also avoids high death rate of the recipient mice based on our previous study(Sun et al., 2021). During irradiation, the brains of the mice were shielded by a lead block to prevent brain damage. Male and female mice were balanced used. To keep the genetic profiles of blood cells identical and the capacity of blood cells to produce Aβ, both irradiated Wt and APOE4 mice were transplanted with the BMCs from age- and sex-matched AD⬝APOE4 mice. The tibias and femurs of the donor mice were harvested and flushed with sterile 0.01M phosphate-buffered saline (PBS) to obtain BMCs. The collected bone marrow cells were incubated with a red blood cell lysis buffer to eliminate erythrocytes and then filtered through a 70-μm cell strainer. The APOE4 and Wt recipient mice were intravenously administered with 10^6^ BMCs in a total volume of 300 μl of sterile PBS via the tail vein (AD⬝APOE4→APOE4; AD⬝APOE4→Wt).

**DNA extraction and analysis of chimerism in blood**

For the purpose of analyzing the chimerism of peripheral blood in the recipient mice, blood cells were collected from the tail vein of Wt, APOE4, and the recipient mice at the age of 12 months. Subsequently, the genomic DNA was purified following the procedures of a commercial DNA extraction kit (TIANGEN, China). Different ratios (0:5, 1:4, 2:3, 3:2, 4:1) of Wt- and APOE4-blood DNA were mixed. Specific primers targeting human APOE4 were employed to amplify the human APOE gene in the samples of both the mixed DNA and the blood DNA of the recipient mice. The PCR products were then loaded onto an agarose gel and photographed using a gel imaging system. The level of the human APOE gene was measured based on the gray value, and polynomial fitting was carried out to explore the relationship between the human APOE gene level and the ratio of the two types of DNA. The ratio of APOE4 and Wt DNA of AD⬝APOE4→Wt recipient mice were calculated according to the fitting formula curve. The chimerism of AD⬝APOE4→Wt recipient mice was equal to the ratio of APOE4 and Wt DNA.

**Blood and brain sampling**

Blood samples were obtained from mice that had been deeply anesthetized with 1% pentobarbital sodium at the age of 12 months. Following centrifugation at 3000 rpm for 10 minutes, the serum was harvested and stored at -80 °C until further utilization. Subsequently, the mice were perfused intracardially with cold 0.01M PBS. The brain hemispheres were meticulously collected, with the right hemisphere being fixed in 4% paraformaldehyde and the left one being promptly frozen and stored at -80 °C for biochemical assays. The fixed brain hemispheres were then transferred to a 30% sucrose solution for dehydration. Afterward, they were sectioned into slices with a thickness of 30 μm using a cryostat. The flash-frozen hemisphere was pulverized into powder in liquid nitrogen. The brain powder was weighed precisely and then divided into three equal portions.

**Histology and quantification**

A series of brain sections that evenly span over the entire brain were selected. These sections were then incubated with a mixture of 3% H₂O₂ and 0.5% Triton X-100 in 0.01M PBS for 15 minutes. Subsequently, they were treated with 3% bovine serum albumin (BSA) and goat serum for 1 hour at room temperature. After this pretreatment, the brain sections were incubated overnight at 4°C with anti-ionized calcium-binding adapter molecule 1 (Iba1) antibody (at a dilution of 1:1000, from Wako, Catalog Number 019-19741 and RRID: AB_839504) and an anti-glial fibrillary acidic protein (GFAP) antibody (at a dilution of 1:1000, from Abcam, Catalog Number ab7260, RRID: AB_305808), respectively. Next, the brain sections were treated with biotin-conjugated anti-rabbit IgG secondary antibody at 37°C for 1 hour, followed by treatment with ABC solution (from Vector). After being thoroughly washed, the samples were incubated with diaminobenzidine substrate and photographed using a microscopy (from ZEISS, Germany). To detect Aβ plaques in the brain, pretreated brain sections were stained with an anti-Aβ (6E10) antibody and then treated with ABC solution and diaminobenzidine substrate. In addition, we also employed Congo Red to detect compact Aβ plaques in the brain according to our previous study(Yu, Yi, et al., 2022). To detect neuronal survival, the brain sections were first treated with an anti-microtubule-associated protein-2 (MAP-2) antibody (at a dilution of 1:1000, from Abcam, Catalog Number ab5392, RRID: AB_2138153) and an anti-neuronal nuclear antigen (NeuN) antibody (at a dilution of 1:1000, from Abcam, Catalog Number ab104224, RRID: AB_10711040). To explore blood-brain barrier (BBB) integrity, brain sections were incubated with an anti-occludin antibody (at a dilution of 1:100, from Invitrogen, Catalog Number 40-6100) and an anti-CD31 antibody (at a dilution of 1:100, from BD Bioscience, Catalog Number 553370, RRID: AB_396660). Subsequently, they were stained with Alexa Fluor 594-labeled anti-mouse IgG secondary antibody and Alexa Fluor 488-labeled anti-rabbit IgG secondary antibody or Alexa Fluor 488-labeled anti-rat IgG secondary antibody and Alexa Fluor 594-labeled anti-rabbit IgG secondary antibody. The quantifications of positive staining in the neocortex and hippocampus were analyzed using ImageJ software (version 1.8.0). The quantification was conducted by a laboratory technician who was blinded to the group information of the slides.

**Western blotting**

Brain powder was utilized to extract proteins with the aid of ice-cold RIPA Lysis buffer supplemented with protease inhibitors and phosphatase inhibitors. The proteins, after being mixed with 5×loading buffer, were loaded onto a 4-20% SDS-polyacrylamide gel for separation. Subsequently, they were transferred onto a nitrocellulose membrane at a current of 250mA for a duration of 50 minutes. The membrane was blocked with quick-block solutions for one hour at room temperature and then incubated overnight at 4°C with a panel of primary antibodies, namely: anti-Disintegrin and metalloproteinase domain-containing protein 10 (ADAM10) antibody (at a dilution of 1:1000, from Abcam, Catalog Number ab1997, RRID:AB_302747), anti-β-site amyloid precursor protein cleaving enzyme 1 (BACE1) antibody (at a dilution of 1:1000, from Abcam, Catalog Number ab108394, RRID:AB_10861218), anti-presenilin (PS1) antibody (at a dilution of 1:1000, from Millipore, Catalog Number MAB5232, RRID:AB_95175), anti-receptor for advanced glycation end products (RAGE) antibody (at a dilution of 1:1000, from Millipore, Catalog Number MAB5328, RRID:AB_95213), anti-low-density lipoprotein receptor-related protein 1 (LRP1) antibody (at a dilution of 1:1000, from Abcam, Catalog Number ab92544, RRID:AB_2234877), anti-pS396-tau antibody (at a dilution of 1:1000, from Abcam, Catalog Number ab109390, RRID:AB_10860822), anti-pT231-tau antibody (at a dilution of 1:1000, from Signalway Antibody, Catalog Number 13381, RRID: Not applicable), anti-Tau antibody (at a dilution of 1:1000, from Proteintech, Catalog Number 10274-1-AP), anti-SYN antibody (at a dilution of 1:1000, from Abcam, Catalog Number ab254349, RRID: AB_2920663), anti-synapse-related proteins including postsynaptic protein-95 (PSD95) antibody (at a dilution of 1:1000, from Millipore, Catalog Number MAB1598, RRID: AB_11212185), anti-synaptosomal-associated protein of 25 kDa (SNAP25) antibody (at a dilution of 1:1000, from Abcam, Catalog Number ab109105, RRID:AB_10887757), anti-occludin antibody (at a dilution of 1:1000, from Invitrogen, Catalog Number 40-6100), and anti-β-actin antibody (at a dilution of 1:2000, from Proteintech, Catalog Number 66009-1-Ig, RRID: AB_2687938). After washed with PBS, the membrane was treated with IRDye 800 CW secondary antibodies and scanned using the Odyssey fluorescent scanner.

**Examination of microglial Aβ phagocytosis**

To reveal the impact of APOE4 on the microglial Aβ phagocytosis, we isolated primary microglia from Wt and APOE4 mice and cocultured with FITC-labeled Aβ. Primary microglia were isolated from the brains of postnatal day 1-3 mice using CD11b microbeads. Briefly, mouse brains were collected and mechanically dissociated into single-cell suspensions by mincing with scalpels followed by 0.25% trypsin and DNase Ⅰ treatment. The cell suspension was then filtered through a 70 μm cell strainer to remove tissue clumps, and centrifuged at 300 × g for 5 min. The pellet was resuspended in FACS buffer (PBS containing 0.2% FBS), incubated with anti-CD11b microbeads (Miltenyi Biotec) at 4°C for 15 min, and passed through a MACS LS column placed on a magnetic separator. After washing the column with FACS buffer to remove unlabeled cells, the CD11b⁺ microglia were eluted by removing the column from the magnet and flushing with buffer. The purified microglia were then centrifuged and collected. Primary microglia were cocultured with 1 μg/mL FITC-labeled Aβ for 1 hour at 37°C in a humidified 5% CO_2_ incubator. Following incubation, cells were collected and subjected to the flow cytometer. The mean fluorescent intensity of FITC-Aβ signal in microglia was measured and analyzed.

**Microdialysis**

Three-month-old male mice Wt and APOE4 mice were subjected to microdialysis experiments (N=4 per group). In brief, a guide cannula (Microbiotech se/AB, Sweden) was stereotactically implanted in the right hippocampus (A/P: − 2.0 mm, M/L: 2.0 mm, D/V: − 2.0 mm) and fixed using binary dental cement. Then, a probe with a 100 kDa cutoff (Microbiotech se/AB, Sweden) was inserted through the guide cannula. The probe was connected to a microdialysis peristaltic pump (CMA, Sweden), which was operated in push–pull mode. Artificial cerebral spinal fluid (ACSF) was introduced at a flow rate of 1 μL/min for interstitial fluid (ISF) collection after intranvenous injection of human Aβ (200μL, 100μg/ml).

**Measurement of blood and brain Aβ levels**

Human Aβ in brain were extracted from brain powder using Tris-Buffered Saline (TBS) and RIPA buffer. Brain and blood human Aβ40 and Aβ42 levels were measured using commercial enzyme-linked immunosorbent assay (ELISA) kit according to the manufacture (Invitrogen, USA). Aβ42 level in brain ISF was also determined by ELISA kit.

**Behavioral Tests**

All 12-month-old experimental mice were subjected to the open-field test, Y maze test, the novel object recognition test (NOR), and the Morris water maze test (MWM) to evaluate their cognitive functions. Prior to the probe test, all mice were transferred to the test room in advance to become familiar with the experimental environment.

**Open-field test**

The open-field test was carried out to assess the locomotor activity ability of the mice. The mice were placed in a box (40cm×40cm×40cm) and allowed to freely explore for 5 minutes. The track paths were recorded using a camera, and the total distance traveled reflected the level of locomotor activity.

**Y maze test**

The Y maze test is used to evaluate the short - term memory of mice. The apparatus consists of a Y-shaped maze with three opaque and identical arms, each positioned at a 120-degree angle to the others. We conducted spontaneous alternation tests and novel arm exploration tests in batches. When assessing spontaneous alternation, the mice were placed in the center of the Y-maze apparatus and permitted to freely explore the arms for 5 minutes. The tracking paths of the mice within the apparatus were recorded. Spontaneous alternation was defined as the total number of alternations × 100% / (the total number of arm entries - 2). During the novel arm exploration test, the mice were first placed in the apparatus for 5-minute exploration with one arm (referred to as the novel arm) blocked. Two hours after the initial exploration of the apparatus, with all arms opened, the mice were placed back into the apparatus and allowed to explore all arms for another 5 minutes. The number of entries into the novel arm and the time spent in the novel arm during the second exploration were analyzed. A higher number of entries into the novel arm or more time spent in it indicates better memory.

**NOR test**

The NOR test is primarily employed to assess recognition memory ability. During the training session, two identical objects were placed at the diagonal corners of the box (40cm×40cm×40cm). The mice were positioned in the center of the box and allowed to freely explore the objects within the box for 5 minutes. After a 2-hour retention period, one of the objects was randomly replaced with a novel object (referred to as the novel one) at the original location. The mice were then returned to the box for free exploration. The time spent exploring and sniffing each object was recorded. The results were presented as the recognition index, which is calculated as [(Time novel)/(Time novel + Time familiar)].

**MWM Test**

The MWM test is an experiment in which experimental animals are forced to swim and learn to find the hidden platforms in the water, mainly used to test the learning and memory abilities of experimental animals in sensing spatial position and direction. The experiment lasted for 6 days, with the first 5 days dedicated to acquisition training and the last day for exploration training. During the first five consecutive days of training, each mouse was placed in a specific quadrant of a pool of water and allowed to swim for 60 seconds to locate an escape platform hidden 1 cm below the water surface for each trial. If the mouse found the platform, it would be kept on it for 3 seconds. If the mouse failed to find the platform within 60 seconds, the experimenter would guide it to the platform and hold it there for 10 seconds. The final trial was conducted on day 6, during which the platform was removed, and the mice were allowed to explore for 60 seconds. The length of time the experimental mouse spent in the target quadrant and the number of times crossing the platform were recorded and analyzed.

Except for the MWM test, after each mouse completed the experiment, the apparatus was cleaned with 75% alcohol to eliminate the influence of odors left by the previous mouse. All performances were tracked using ANY-maze software (USA). The testing order was randomly assigned by the ANY-maze software, and the experimental performers were blinded to the grouping information.

**Statistical analysis**

The data are presented in the form of means ± standard errors of the mean (SEMs). All statistical analyses were carried out using SPSS 23.0 software (Chicago, USA). The statistical graphs were generated by GraphPad Prism (version 10.1.2). Initially, the Shapiro-Wilk test was utilized to assess whether the data conformed to a normal distribution. Subsequently, if the data adhered to a normal distribution, independent t-tests were employed to evaluate the comparisons between two groups. Conversely, in cases where the data did not follow a normal distribution, a non-parametric statistical method, namely the Mann‒Whitney U test, was utilized. For the escape latency data in the MWM test, a repeated measures analysis of variance (ANOVA) was implemented. A P value less than 0.05 was regarded as statistically significant.

**References**

Sun, H. L., Chen, S. H., Yu, Z. Y., Cheng, Y., Tian, D. Y., Fan, D. Y., . . . Wang, Y. J. (2021). Blood cell-produced amyloid-β induces cerebral Alzheimer-type pathologies and behavioral deficits. *Mol Psychiatry, 26*(10), 5568-5577. doi:10.1038/s41380-020-0842-1

Yu, Z. Y., Xu, M. Y., Liu, Z. H., Zeng, G. H., Fan, H., Tan, C. R., . . . Wang, Y. J. (2022). Effects of Chemotherapy on Neuroinflammation, Neuronal Damage, Neurogenesis, and Behavioral Performance in Bone Marrow Transplantation Recipient Mice. *Neurotox Res, 40*(2), 585-595. doi:10.1007/s12640-022-00494-7

Yu, Z. Y., Yi, X., Wang, Y. R., Zeng, G. H., Tan, C. R., Cheng, Y., . . . Liu, Y. H. (2022). Inhibiting alpha1-adrenergic receptor signaling pathway ameliorates AD-type pathologies and behavioral deficits in APPswe/PS1 mouse model. *J Neurochem, 161*(3), 293-307. doi:10.1111/jnc.15603

**Supplementary figures**


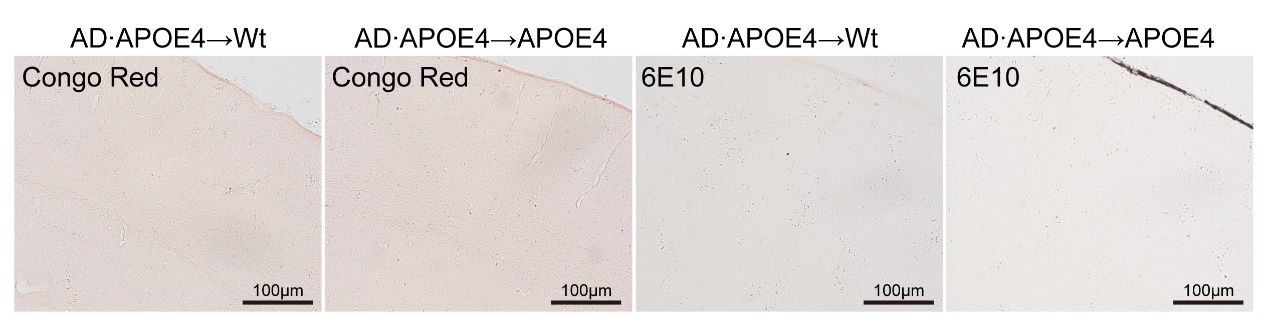


**Figure S1. Effect of APOE4 on cerebral Aβ deposition after BMT.**

Representative images of 6E10 staining and Congo Red staining of the brain. Scale bar is 100μm. AD⬝APOE4→Wt mice denotes that Wt mice received bone marrow cells from APP/PS1 mice carrying humanized APOE4 gene. AD⬝APOE4→APOE4 mice denotes that APOE4 mice received bone marrow cells from APP/PS1 mice carrying humanized APOE4 gene. BMT denotes bone marrow transplantation.

**
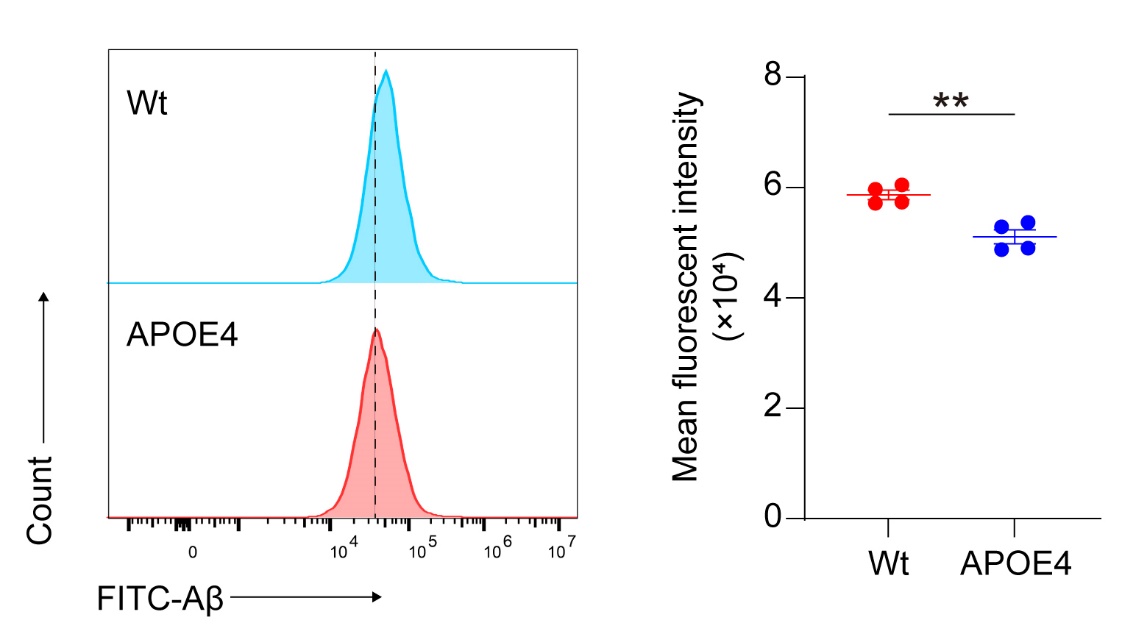
**

**Figure S2. Impact of APOE4 on microglial Aβ phagocytosis.**

Representative flow-cytometric image of microglial Aβ phagocytosis and comparison of mean fluorescent intensity of FITC-Aβ in microglia (N=4 per group). ** Denotes P<0.01.

**
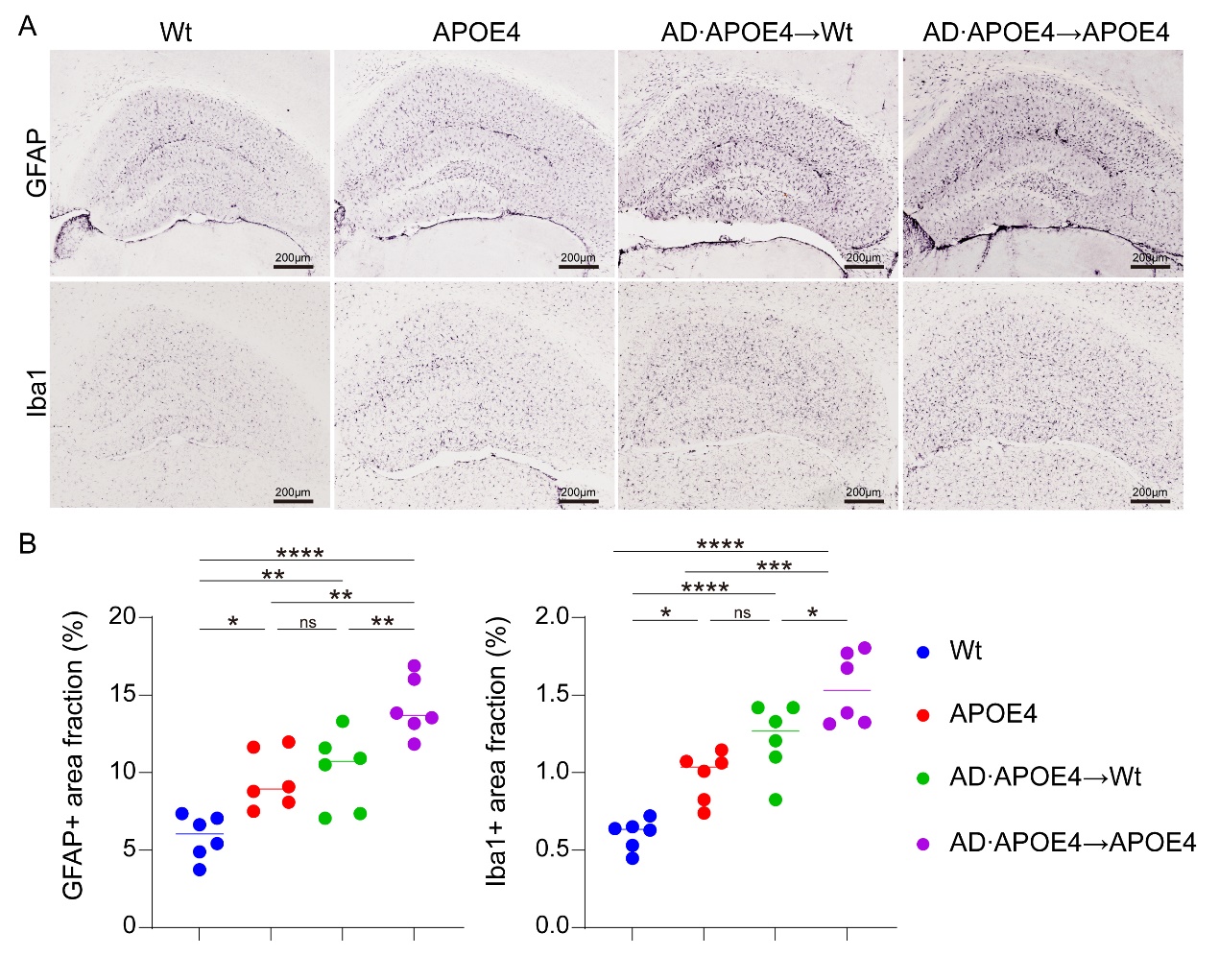
**

**Figure S3. Microgliosis and astrogliosis induced by blood-derived Aβ and APOE4.**

A. Representative immunochemistry images of Iba1 staining and GFAP staining. B. Comparisons of area fraction of Iba1+ and GFAP+ staining and hippocampus between Wt, APOE4, AD⬝APOE4→Wt mice and AD⬝APOE4→APOE4 mice (N=6 per group). * Denotes P<0.05; ** denotes P<0.01; *** denotes P<0.001; **** denotes P<0.0001. The scale bar in A is 200μm.
